# Morphological characterization reveals new insights into giant cell development of *Meloidogyne graminicola* on rice

**DOI:** 10.1007/s00425-022-03852-z

**Published:** 2022-02-19

**Authors:** Yongrui Niu, Liying Xiao, Janice de Almeida-Engler, Godelieve Gheysen, Deliang Peng, Xueqiong Xiao, Wenkun Huang, Gaofeng Wang, Yannong Xiao

**Affiliations:** 1 Key Laboratory of Plant Pathology of Hubei Province, College of Plant Science and Technology, Huazhong Agricultural University, Wuhan, 430070 China; 2 INRAE, CNRS, ISA, Université Céte d’Azur, 06903 Sophia Antipolis, France; 3 Department of Biotechnology, Faculty of Bioscience Engineering, Ghent University, 9000 Ghent, Belgium; 4 State Key Laboratory for Biology of Plant Diseases and Insect Pests, Institute of Plant Protection, Chinese Academy of Agricultural Science, Beijing, 100193 China

**Keywords:** Cytoplasm density, Giant cells, *Meloidogyne graminicola*, *Oryza sativa*, Polarized expansion, Three-dimensional structures

## Abstract

**Main conclusion:**

Three types of nematode-feeding sites (NFSs) caused by *M. graminicola* on rice were suggested, and the NFS polarized expansion stops before the full NFS maturation that occurs at adult female stage.

**Abstract:**

Root-knot nematodes, *Meloidogyne* spp., secrete effectors and recruit host genes to establish their feeding sites giant cells, ensuring their nutrient acquisition. There is still a limited understanding of the mechanism underlying giant cell development. Here, the three-dimensional structures of *M. graminicola*-caused nematode-feeding sites (NFSs) on rice as well as changes in morphological features and cytoplasm density of the giant cells (GCs) during nematode parasitism were reconstructed and characterized by confocal microscopy and the Fiji software. Characterization of morphological features showed that three types of *M. graminicola*-caused NFSs, type I–III, were detected during parasitism at the second juvenile (J2), the third juvenile (J3), the fourth juvenile (J4) and adult female stages. Type I is the majority at all stages and type II develops into type I at J3 stage marked by its longitudinal growth. Meanwhile, NFSs underwent polarized expansion, where the lateral and longitudinal expansion ceased at later parasitic J2 stage and the non-feeding J4 stage, respectively. The investigation of giant cell cytoplasm density indicates that it reaches a peak at the midpoint of early parasitic J2 and adult female stages. Our data suggest the formation of three types of NFSs caused by *M. graminicola* on rice and the NFS polarized expansion stopping before full NFS maturation, which provides unprecedented spatio-temporal characterization of development of giant cells caused by a root-knot nematode.

**Supplementary Information:**

The online version contains supplementary material available at 10.1007/s00425-022-03852-z.

## Introduction

Root-knot nematodes (RKNs), *Meloidogyne* spp. are soilborne pathogens of crops causing tremendous yield losses, and have been determined to be one of top ten plant parasitic nematodes based on their scientific and economic importance (Jones et al. 2013). The rice RKN, *M. graminicola*, is a major threat to rice production (Mantelin et al. 2017), and serious damage has been reported on rice plants in China (Song et al. 2017; Tian et al. 2017; Wang et al. 2017; Xie et al. 2019; Ju et al. 2021; Liu et al. 2021). Hence, uncovering the parasitic mechanisms of *M. graminicola* is important to develop new strategies for control of this nematode.

The complete infection process of *M. incognita* in roots of *Arabidopsis thaliana* has been investigated by Wyss et al. (1992). The pre-parasitic second-stage juveniles of *M. incognita* invade plant roots in the elongation zone by penetrating and destroying epidermal cells. They then migrate intercellularly down to the meristem, turning round to enter into the vascular tissue. Successively, nematodes induce root swellings, named galls, characterized by the induction of vascular parenchyma asymmetric cell division (de Almeida-Engler et al. 2015). These nematode-feeding sites (NFSs), composed of asymmetrically shaped giant cells (GCs) embedded in the gall, are likely induced by injection of a cocktail of secreted effectors (Wyss et al. 1992; Berg et al. 2008). NFSs are unique organs with specific cell wall signatures, presenting high metabolic activities and functioning as the nutrient supply throughout the nematode life cycle (Grundler and Hofmann 2011; Bozbuga et al. 2018; Meidani et al. 2019; Sato et al. 2019). Different from the NFSs caused by cyst nematodes that are achieved by cell wall dissolution of neighboring cells (Sobczak and Golinowski 2009), GCs caused by *Meloidogyne* spp. originate from karyokinesis without cytokinesis evidenced by previous investigations of cell wall stubs, expression of marker genes involved in mitosis, assessment of DNA content in GCs as well as the observation of spindles formed by microtubules in GCs (de Almeida-Engler et al. 1999; Banora et al. 2011; de Almeida-Engler and Gheysen 2013; Escobar et al. 2015).

In the last decade, empirical data on molecular mechanisms of GC development are accumulating. For instance, *M. incognita* modulates the host mitotic cell cycle by recruiting the host cell inhibitors of the *KRP6* family and genes controlling the S-phase like *ABAP1* in *Arabidopsis* (Vieira and de Almeida-Engler 2015). As well, the *WEE1* kinase gene of *Arabidopsis* is induced by *M. incognita* infection to function in checkpoint control activation in the GCs (Cabral et al. 2020). However, there are still important questions on GC development that need to be answered, in particular how their growth progresses at each parasitic nematode developmental stage (J2, J3, J4 until adult female). Using more rigorous sampling methods, instead of analyzing certain time points that contain mixed developmental stages of both GCs and nematodes, more reliable cellular analyses can be performed. Thus, performing serial analyses which cover all GC developmental stages associated to identified nematode stage development, we expect to shed some light on the conundrums. With this aim, extensive investigations of GC sections and three-dimensional (3D) reconstruction of root-knot nematode (RKN)-caused NFSs during four nematode developmental stages were performed here to elucidate the developmental progression of NFSs caused by *M. graminicola* in the important crop host, *Oryza sativa*.

3D reconstruction of NFSs induced by *M. javanica* in the model host *A. thaliana* was first conducted by Cabrera et al. (2015) based on serial sections of NFSs. As well, Cabrera et al. (2018) described a method that allows the observation of intact NFSs in entire galls and 3D reconstruction of the intact NFSs. To extensively characterize NFS structures at parasitic J2 stage, we divided this stage into four substages with the consideration of the initial development of NFSs at the parasitic J2 stage. Finally, 3D structures of NFSs caused by *M. graminicola* on rice at each developmental stage were reconstructed and the expansional progression as well as the cytoplasm density of NFSs along the development progression of *M. graminicola* were described. Thus, our data provide a new interpretation of the developmental mechanism of NFSs caused by *M. graminicola* on rice.

## Materials and methods

### Plant and nematode culture

The nematode *M. graminicola* was isolated from rice in Haikou, Hainan Province, China, and was cultured on rice (*Oryza sativa* cv. Nipponbare) in controlled environmental conditions (28 °C under a 12-h/12-h light/dark regime). For all the following experiments, the rice cultivar, *O. sativa* cv. Nipponbare, was used and rice plants were grown under the same controlled environmental conditions.

### Nematode inoculation

Rice seeds (approximately 25 seeds per plate) were germinated in plates carrying 3 layers of seed germination paper, and 7-day-old seedlings cultured in the plates were directly inoculated with approximately 150 s stage juveniles (J2s) of *M. graminicola* per plate.

### Fixation and clearing of gall samples

The fixation and clearing of gall samples were performed according to the method described by Cabrera et al. (2018) with some modifications. First, *M. graminicola*-caused galls with one to three nematodes per galls were hand-dissected at 1 day post-inoculation (dpi), 3 dpi, 7 dpi and 10 dpi, respectively, and collected immediately in 50 mM sodium-phosphate buffer pH 7 (PBS) at room temperature. Samples were then fixed in the same buffer containing 3% (*v*/*v*) glutaraldehyde under vacuum for 15 min, refreshed twice and kept overnight at 4 °C. Fixed galls were then rinsed twice for 5 min with PBS and were sequentially dehydrated for 20 min each in 30%, 50%, 70%, 90% (*v*/*v*) ethanol in PBS with the modification of carrying out each process under vacuum and repeating each treatment twice. Finally, dehydrated galls were rinsed twice for 20 min with pure ethanol under vacuum, and kept overnight in pure ethanol at 4 °C. The subsequent clearing process was performed according to Cabrera et al. (2018) with the modification of carrying out each process under vacuum and repeating each treatment twice. Finally, galls were maintained in 100% BABB [benzyl alcohol; Sigma 402834; St. Louis, MO, USA)/benzyl benzoate (Sigma B6630); mixed in a 1:2 proportion (*v*/*v*)] 2 days at 4 °C.

### Confocal microscopy analysis of nematode-feeding sites inside galls

Cleared *M. graminicola* induced galls were observed under a Leica TCS SP8 laser scanning confocal microscope (Leica, Wetzlar, Germany) and the serial optical sections of each *M. graminicola*-caused NFS that is composed of all the giant cells (GCs) caused by a nematode were obtained. The green auto-fluorescence generated by the glutaraldehyde was recovered for image capture in the range of 500–600 nm as described (Cabrera et al. 2018).

### Image analysis

The software Fiji was used to analyze the image stacks that were obtained by laser scanning confocal microscopy (Schindelin et al. 2012). For three-dimensional (3D) reconstructions of NFSs, the plugin 3D View was used with the pre-established parameters except of the resampling factor being 1, which was used to facilitate the distinction of boundaries as well as the lateral and longitudinal zones of *M. graminicola*-caused NFSs in the following analyses.

For volume measurements of each NFS, the total NFS area of its complete image stack was first measured using the freehand selection tool of Fiji, where the largest expanded area of a NFS was identified to be the largest NFS area among its complete serial sections. Then, the total NFS area was multiplied by the voxel depth to obtain the volume of an NFS. For measurements of both lateral and longitudinal areas of NFSs, the two terms lateral and longitudinal zones of NFSs were artificially defined here. The lateral zone of a NFS was that being perpendicular to the nematode head, and its longitudinal zone was that with the largest projected area and being perpendicular to its lateral zone. The plugin, Volume Viewer, was used to obtain images of both the lateral and longitudinal zones. The model of Volume was used for the GC with high cytoplasm density and the mode of Projection for that with low cytoplasm density. The lateral and longitudinal areas were measured according to that of NFS area. In addition, the following two statistical formulas were developed to assess the differences between the lateral and longitudinal area of NFSs:

$$ \begin{gathered} \overline{a}\left( \% \right) = {1}00 \times \left( {{\text{longitudinal area}} - {\text{lateral area}}} \right)/{\text{lateral area}}; \hfill \\ \overline{e}\left( \% \right) = {1}00 \times \left( {{\text{lateral area}} - {\text{longitudinal area}}} \right)/{\text{longitudinal area}}. \hfill \\ \end{gathered} $$

For quantifying cytoplasm density of GCs caused by *M. graminicola* on rice, the GCs with the largest area were used and the mean gray value of them were measured using the freehand selection tool of Fiji, using root cortex cells as an internal control for normalization. Then, the relative cytoplasm density of each GCs (mean gray value of GCs/mean gray value of cortex cells) was calculated.

### Identification of nematodes’ developmental stages inside galls

After confocal microscopy analysis, *M. graminicola* juvenile nematodes were hand-dissected from the galls under a stereomicroscope and their developmental stages were characterized according to Triantaphyllou and Hirschmann (1960) and Garcia-Martinez (1982). The body shape, position of stylet and number of layers of cuticle were the morphological identification indexes of the classification of nematode developmental stages.

### Statistical analysis

Experimental data were analyzed using the SPSS v. 30.0 (SPSS Inc.), Student’s *t *test was applied for pairwise comparisons, or one-way ANOVA with post hoc Tukey HSD test for multiple comparisons of group means. Values with *P* < 0.05, *P* < 0.01 or *P* < 0.001 were considered statistically significant.

## Results

### Morphology of different developmental stages of *M. graminicola*-caused nematode-feeding sites on rice

**Fig. 1:**
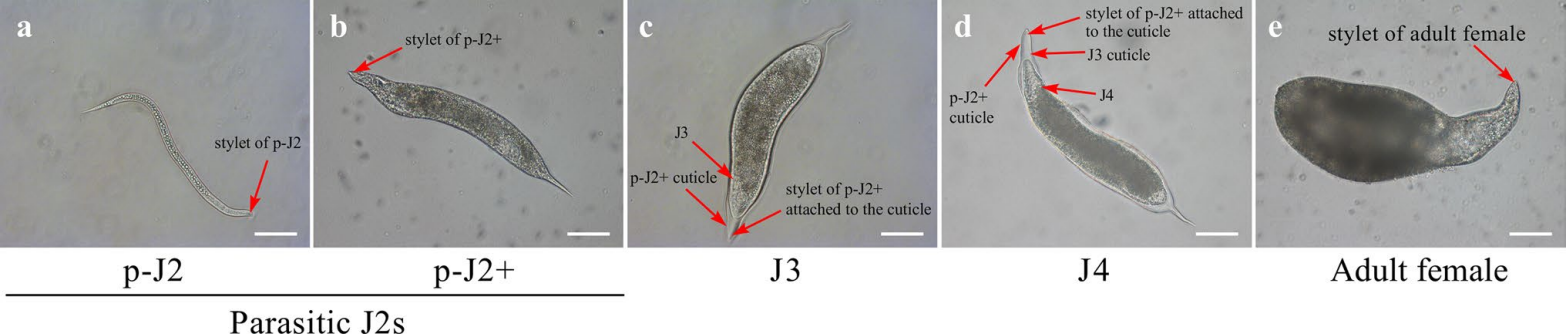
Morphologies of nematodes at different developmental stages. Developmental stages analyzed were named: early (p-J2) and later (p-J2+) parasitic J2 stages as well as J3, J4 and adult female (adult) stages were identified according to the body shape of *M. graminicola* and its stylet and cuticle. Bar = 100 μm

**Fig. 2:**
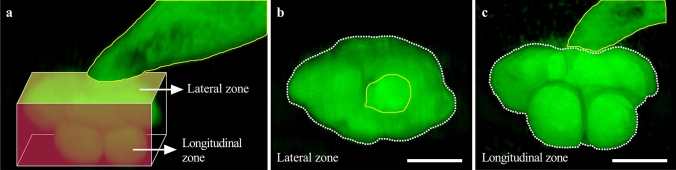
Identifications of lateral and longitudinal zones of NFSs. Lateral and longitudinal zones of a *M. graminicola*-caused NFS on rice were indicated in the 3D structure (**a**) and 2D pictures (**b** and **c**) of the NFS. Yellow lines surround nematodes, and white dotted lines surround NFSs. Bar = 80 μm

To assess changing morphologies of developing *M. graminicola*-caused NFSs that are composed of GCs on rice, a total of 167 NFSs at different stages were analyzed (Table S1). The developmental stages of the NFSs were identified according to that of the stage of the feeding nematode (Fig. 1). *M. graminicola* at early parasitic J2 stage shows a linear body shape and holds a stylet used to secrete molecules and to feed (Fig. 1a), while at later parasitic J2 stage, the body increases significantly in volume (Fig. 1b). Both J3 and J4 have no stylet and show similar body shapes as later parasitic J2 stage, where J3 stay enclosed in the old parasitic J2 cuticle and J4 in both the old parasitic J2 and J3 cuticles (Fig. 1c, d). Adult females of *M. graminicola* are saccate pear-shaped and new stylets can be observed at the head (Fig. 1e). We analyzed here NFSs containing from early to later parasitic J2 (35, 22), J3 (32), J4 (27) stages and adult females (51) (Table S1). The lateral and longitudinal areas and volumes of the NFSs at each stage were measured (Fig. 2, Table S1), except for the early parasitic J2 stage where only the volumes were assessed (Table S1).

### Three types of *M. graminicola*-caused nematode-feeding sites on rice can be distinguished

**Fig. 3:**
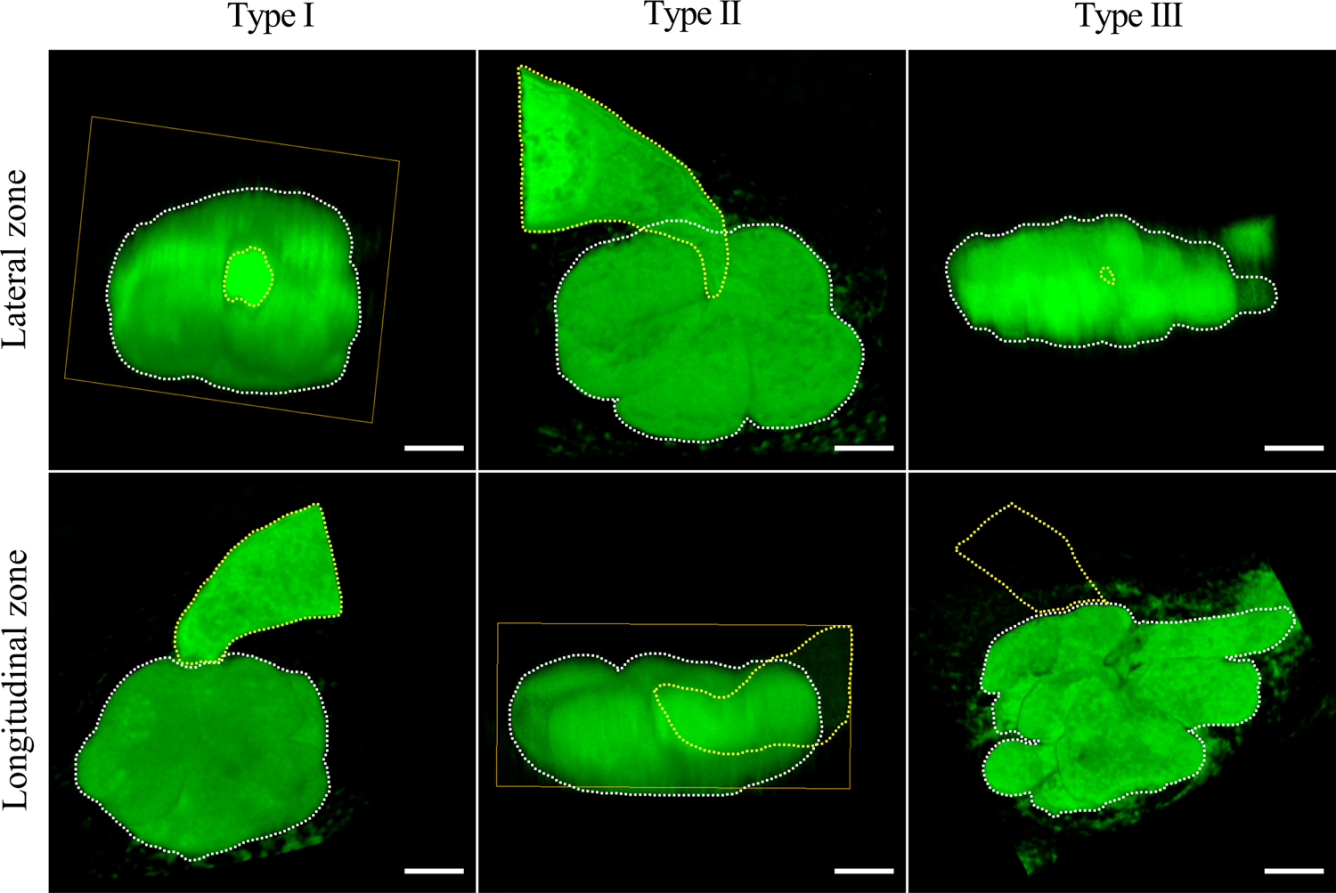
Three-dimensional structures of three types of NFSs. Lateral and longitudinal zones of type I and II at adult female stage as well as type III at J3 stage were characterized. Yellow and white dotted lines indicate nematodes and NFSs, respectively. Yellow rectangles indicate artificial cross-sections when performing 3D reconstruction of *M. graminicola*-caused NFSs. Bar = 50.0 μm

Based on the difference in 3D structures of *M. graminicola*-caused NFSs on rice at different developmental stages, data of lateral and longitudinal areas of *M. graminicola*-caused NFSs were measured and compared, leading to the classification of three types of *M. graminicola*-caused NFSs: type I ($\overline{a}$ < 45.0 and $\overline{e}$ < 45.0), type II ($\overline{e}$ ≥ 45.0), and type III ($\overline{a}$ ≥ 45.0) (Fig. 3, Video S1–S3). According to the definition of the types, type I shows a similar size for its lateral and longitudinal areas (Fig. 3, Video S1). However, type II has a significantly larger lateral area than its longitudinal area, while it is opposite for type III (Fig. 3, Video S2–S3). The correlation between the largest expanded area and volume of *M. graminicola*-caused NFSs at the four stages tested was low with the correlation indexes (*R*^2^) ranging from 0.4848 to 0.7449 (Fig. S1), which implies a difference in 3D structures of NFSs caused by *M. graminicola* on rice.

### Nematode-feeding sites of type II at later parasitic J2 stage develop into type I at J3 stage by longitudinal expansion

**Fig. 4:**
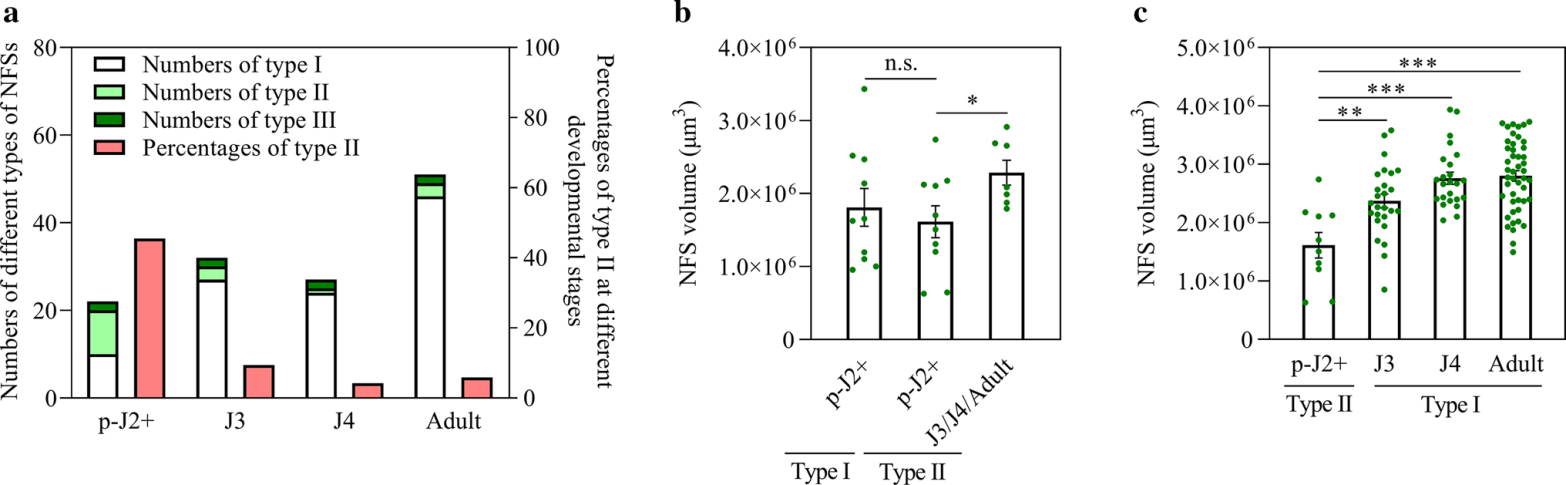
Evidences of the type II of *M. graminicola*-caused NFSs developed into the type I. **a** Numbers of three types of NFSs caused by *M. graminicola* on rice and percentages of type II in all three types at later parasitic J2 stage (p-J2+), J3, J4 and adult female stages were assessed. **b** Differences in NFS volume between type I and type II at p-J2+ and between type II at p-J2+ and its later stages, J3, J4 and adult female stages were evaluated. **c** Difference in NFS volume between type II at p-J2+ and type I at J3, J4 and adult female stage was evaluated. Values are means ± SE. *, ** and *** indicate significant differences (Student’s *t* test) at *P* < 0.05, *P* < 0.01 and *P* < 0.001, respectively. n.s. means not significant

Of the 22 analyzed NFSs at the later parasitic J2 stage, 10 were found to be of type I, 10 of type II and only 2 of type III (Fig. 4a). While the type III was equally rare at all parasitic stages, a shift from type II to type I was seen at J3 stage with the percentage going down from 45.4 at later parasitic J2 stage to 9.4 at J3 stage (Fig. 4a). There are three possibilities that could lead to the percentage reduction of type II at J3 stage, including the development of type II into type I at later parasitic J2 stage or at J3 stage or by the further expansion of type II as well as the cell death of type II at J3 stage. To assess the possibility that type II develops into type I at later parasitic J2 stage by its further expansion, NFS volume of type I and type II at this stage were compared. A non-significant difference was found in NFS volume between type I and II at later parasitic J2 stage (Fig. 4b), indicating the synchronous development of type I and II at this stage that excludes the possibility of type II developing into type I at later parasitic J2 stage. Then, the possibility of the cell death of type II at J3 stage was evaluated by comparing NFS volume of type II at later parasitic J2 with that at the later stages (including J3, J4 and adult female stages). The results showed that NFS volume of type II at the later stages increased significantly compared with that at later parasitic J2 stage with the average NFS volume increasing from approximately 1.6 × 10^6^ μm^3^ at later parasitic J2 stage to approximately 2.3 × 10^6^ μm^3^ at the later stages (Fig. 4b), suggesting the continuous development of type II at J3, J4 and adult female stages. In addition, no dead NFSs were detected among all the gall samples at each stage (data not shown). These findings elucidate that the decrease of type II at J3 stage does not result from the cell death of type II at J3 stage. For testing the third possibility of type II developing into type I at J3 stage by the continuous expansion of type II, NFS volume of these two types at later parasitic J2 stage as well as NFS volume of type II at later parasitic J2 stage and type I at the later stages, including J3, J4 and adult female stages, were compared. Results indicated that type II at later parasitic J2 stage was found significantly smaller than that of type I at its later stages, J3, J4 and adult female stages (Fig. 4c), which makes it reasonable that type II at later parasitic J2 stage develops into type I at J3 stage by the further expansion of type II.

**Fig. 5:**
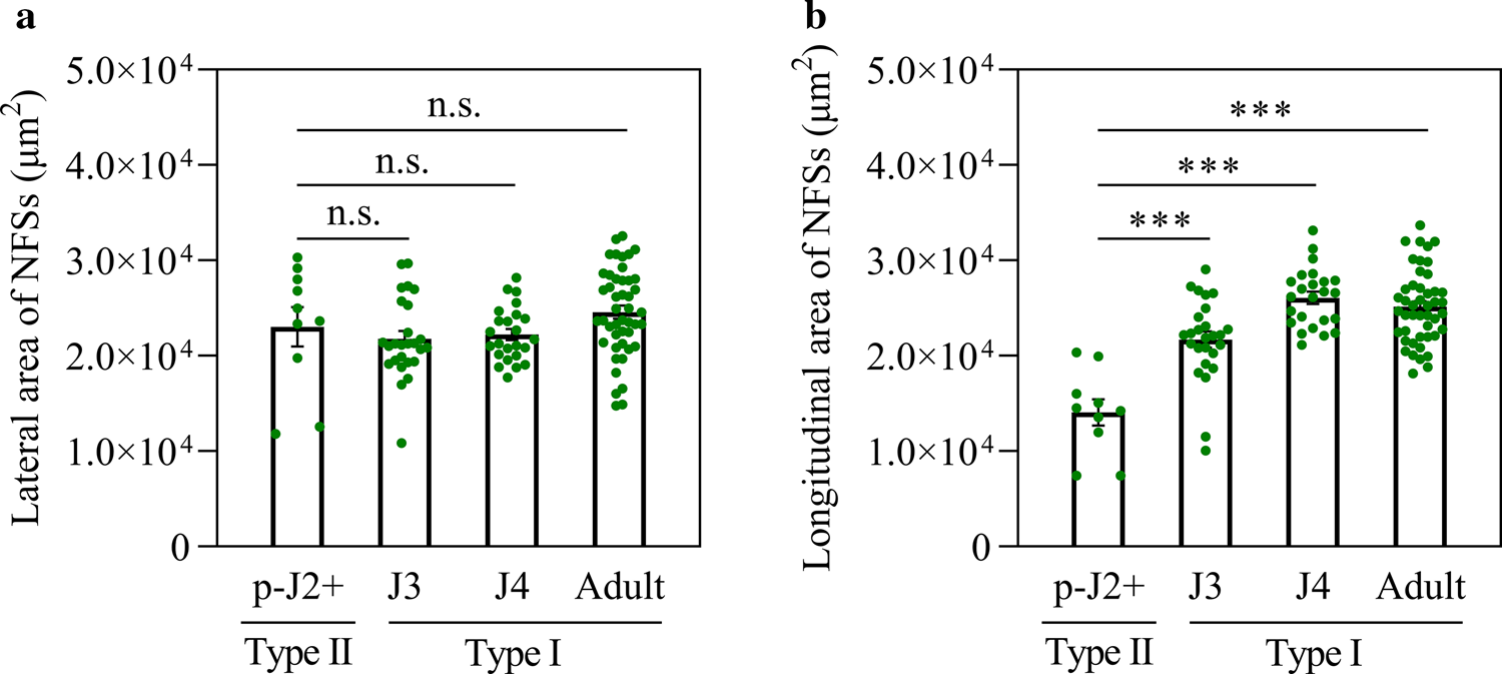
Comparisons of lateral and longitudinal areas of NFS type II with type I at later stages of parasitic J2. Differences in both lateral (**a**) and longitudinal (**b**) areas of NFSs between type II at later parasitic J2 stage (p-J2+) and type I at J3, J4, and adult female (adult) stage were estimated, respectively. Values are means ± SE. *** indicate significant differences (Student’s *t* test) at *P* < 0.001. n.s. mean no significant at *P* < 0.05

To uncover the potential mechanism of type II developing into type I at J3 stage, differences in both the lateral and longitudinal areas of type II at parasitic J2 stage and type I at J3, J4 and adult female stages were estimated. No significant difference in the lateral area was observed between type II at later parasitic J2 stage and type I at the later stages, J3, J4 and adult female stages (Fig. 5a), but the longitudinal area was highly significantly increased since J3 stage (Fig. 5b). It is postulated that type II at early parasitic J2 stage develops into type I at J3 stage through its longitudinal expansion.

### Polarized expansion of *M. graminicola*-caused nematode-feeding sites ceases at J4 stage

**Fig. 6:**
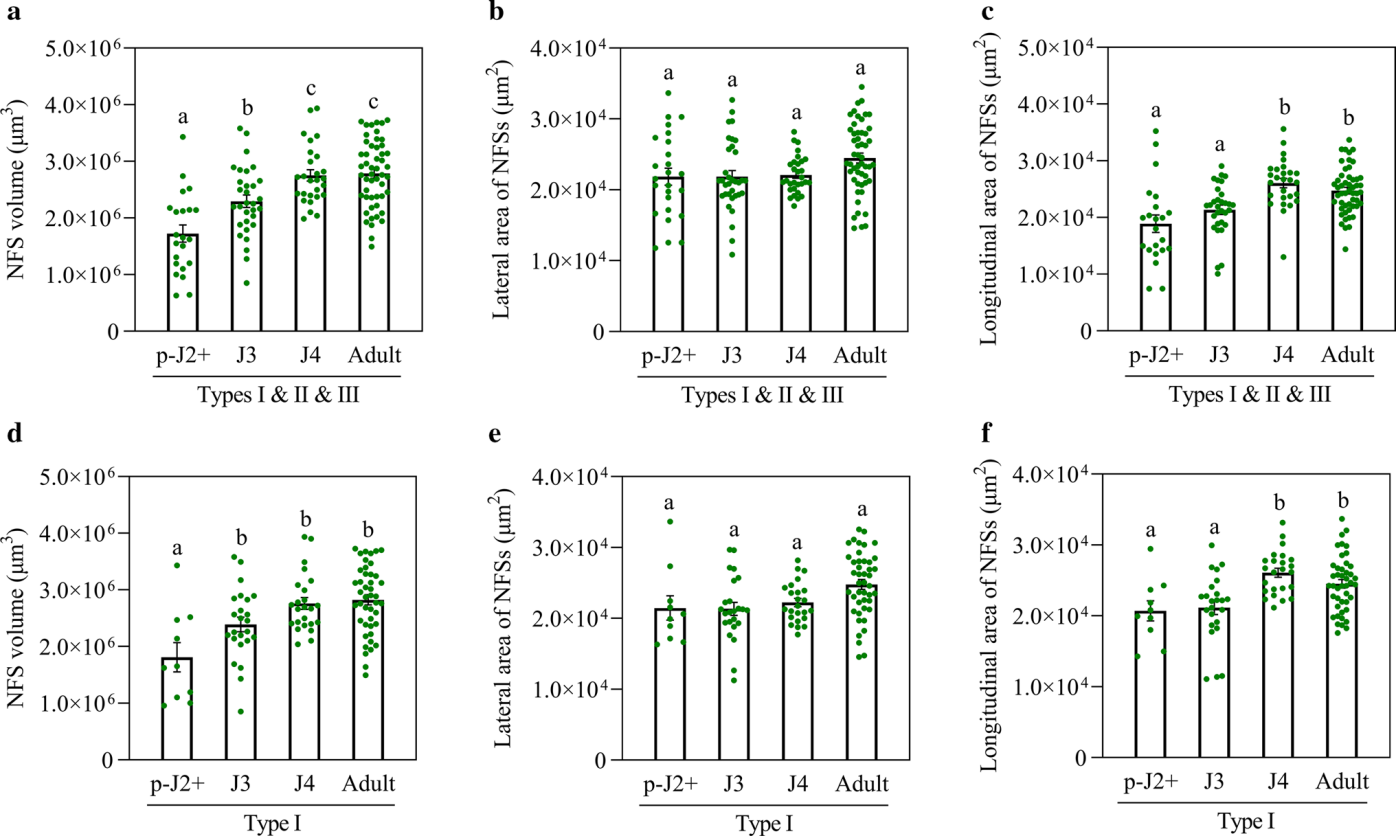
Differences in volume, lateral and longitudinal areas of *M. graminicola*-caused NFSs among different developmental stages. The volume as well as lateral and longitudinal areas of both all the three types (**a**–**c**) and the type I (**d**–**f**) of *M. graminicola*-caused NFSs among later parasitic J2 (p-J2 +), J3, J4 and adult female (adult) stages were evaluated. Values are means ± SE. The different lowercase letters indicate significant differences (one-way ANOVA with post hoc Tukey HSD test) at *P* < 0.05

The volumes, lateral and longitudinal areas of *M. graminicola*-caused NFSs among later parasitic J2 stage as well as J3, J4 and adult female stages were compared to decipher the expansion progression of these NFSs. First, the difference in NFS volume among the four stages was compared. Result showed that the NFS volume increased until J4 stage without taking into consideration NFS types (Fig. 6a), and until J3 stage for type I (Fig. 6d). These data imply that the expansion of NFSs caused by *M. graminicola* on rice ceases at J4 stage. Differences in both lateral and longitudinal areas were also assessed to evaluate the potential polarized expansion of the *M. graminicola*-caused NFSs. Data indicated that the lateral area did not significantly change after the later parasitic J2 stage neither for all the three types (types I, II and III) NFSs nor for only type I (Fig. 6b, e), but an increase of longitudinal area was observed until J4 stage for the two groups of NFSs (Fig. 6c, f). This demonstrates that the lateral expansion of NFSs stops at later parasitic J2 stage, whereas the longitudinal expansion stops at J4 stage. Based on these data, it was proposed that *M. graminicola*-caused NFSs on rice undergo a polarized expansion until J4 stage.

### Cytoplasm densities of *M. graminicola*-caused GCs reach a peak at both early parasitic J2 and adult female stages

**Fig. 7:**
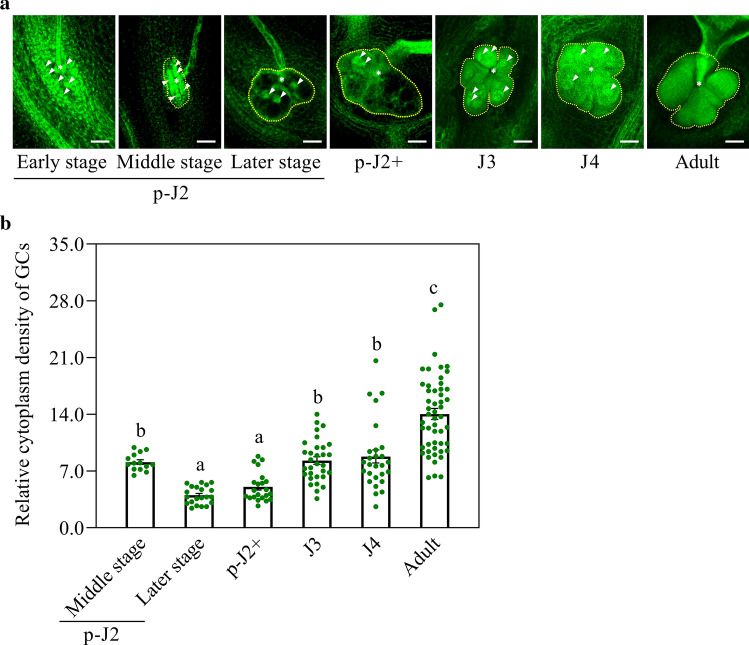
Difference in cytoplasm density of *M. graminicola*-caused GCs among different developmental stages. Sections of GCs caused by *M. graminicola* on rice at the early, middle and later of early parasitic J2 stage (p-J2) as well as later parasitic J2 (p-J2+), J3, J4 and adult female (adult) stages were photographed by confocal microscopy (**a**). The relative cytoplasm density of *M. graminicola*-caused GCs were measured by the software Fiji, using cortex cells as an internal control for normalization (**b**). Yellow dotted lines are NFSs, asterisks are the nematode heads and white arrowheads nuclei. Voxel depth of the sections at early, middle and later of early parasitic J2 stage as well as later parasitic J2, J3, J4 and adult female stage was 2.79 μm, 1.40 μm and 1.44 μm as well as 1.85 μm, 1.50 μm, 2.21 μm and 1.91 μm, respectively. Bar = 50.0 μm. Values are means ± SE. The different lowercase letters indicate significant differences (one-way ANOVA with post hoc Tukey HSD test) at *P* < 0.05

Cytoplasm density of 173 M*. graminicola*-caused GCs was investigated during their development, of which 41 were at early parasitic J2 stage. Three kinds of GCs caused by *M. graminicola* on rice were found at the early parasitic J2 stage according to the differences in cytoplasm density and boundary (the GC walls) of GCs. The first concerned the very early GCs (1 dpi) with remarkable nuclei, and the surrounding fluorescing nuclei from cells neighboring the GCs (Fig. 7a, Fig. S2). The second were GCs with high cytoplasm density and distinguishable nuclei and boundaries (Fig. 7a, Fig. S3). Similar to the second, the third type of GCs also contained detectable nuclei and distinguishable boundaries, but low cytoplasm density with few strongly stained nuclei in their neighboring cells (Fig. 7a, Fig. S4). To compare GC development between the second and the third kind of *M. graminicola*-caused GCs, volumes of them were measured and compared. It was shown that the second kind of GCs were significantly smaller than the third (Fig. S5), implying that GC expanded as NFS matured. For *M. graminicola*-caused GCs at later parasitic J2 stage as well as J3 and J4 stages, distinguishable GC boundaries as well as enlarged GC nuclei were clearly visualized, and cytoplasm density of the GCs tended to increase along their development (Fig. 7a, Fig. S6–S8). GCs at adult female stage showed a more homogeneous green fluorescence (Fig. 7a, Fig. S9) suggesting the highest cytoplasm density of GCs at this stage compared to that at the early stages of infection like parasitic J2 and the non-feeding J3 and J4 stages. This result was further validated by the quantification of relative cytoplasm density of *M. graminicola*-caused GCs on rice to that of their neighboring cells at each stage. The relative cytoplasm density of *M. graminicola*-caused GCs at middle of early parasitic J2 stage was significantly higher than that at later of parasitic J2 stage with the relative cytoplasm density being 8.1 and 4.0, respectively (Fig. 7b). Later, the relative cytoplasm density of the GCs increased at J3 stage with the relative density being 8.3 (Fig. 7b), followed by a significant increase at adult female stage, leading to the highest cytoplasm density at adult female stage with the relative cytoplasm density being 14.0 (Fig. 7b).

## Discussion

Sedentary parasitic nematodes such as *Meloidogyne* spp. invade plant roots to induce NFSs by secreting a number of effectors through their stylets leading to the induction of GCs. These GCs will then provide nutrients for the developing nematode until the end of their life cycle (Kyndt et al. 2013). Although developmental structures of these NFSs have been considerably studied (Vieira and Gleason 2019; Cabral et al. 2020; Olmo et al. 2020), we still lack a defined understanding on how NFSs develop during infection. Here, we address the NFS architecture and development by reconstructing 3D structures of NFSs caused by *M. graminicola* on rice during various developmental stages: parasitic J2, J3, J4 and adult female stages, by applying the imaging method reported by Cabrera et al. (2018). This method allows an observation of NFSs via fluorescence of the fixed tissue, making it possible to characterize the intact 3D grouped GC structures. Green fluorescence signals detected in NFSs fixed by glutaraldehyde result from the extensive protein cross-linking by glutaraldehyde (Migneault et al. 2004; Cabrera et al. 2018). Systematic investigation of 3D structures of NFSs at various stages allowed us to characterize their developmental progression. In the previous study, gall samples were harvested at different time points (Cabrera et al. 2015), implying that GCs within gall samples analyzed are at mixed developmental stages, thus, leading to an ambiguous description of their 3D structures during development. To overcome this shortcoming, different developmental stages of *M. graminicola* were precisely identified in NFSs in rice roots during parasitism. More precisely, morphological features of NFSs were first characterized during *M. graminicola* parasitism after which the nematodes were isolated from the galls for identification of their developmental stage based on morphological features (Triantaphyllou and Hirschmann 1960; Garcia-Martinez 1982). Data of 3D reconstruction of NFSs caused by *M. graminicola* on rice showed differences in their structures at each stage tested. To describe differences in 3D structure and analyze the developmental relationships, two terms named lateral and longitudinal areas of NFSs were introduced and compared. These resulted in the observation of three types of *M. graminicola*-caused NFSs on rice, named here type I, type II and type III, according to their measurements. *M. javanica*-caused NFSs on *Arabidopsis* with different 3D structures characterized by one-dome or two-dome shape have also been reported (Cabrera et al. 2015). A high correlation was described between the volume and the largest expanded area of NFSs caused by *M. javanica* on *Arabidopsis* (Cabrera et al. 2015). Differently, this was not observed for NFSs caused by *M. graminicola* on rice. This could be explained by differences in 3D structure among these two kinds of NFSs on rice and *Arabidopsis*. Using the largest NFS expanded area to evaluate their volume has shown here to be unreliable at least when estimating the NFS volume in the rice–*M. graminicola* interaction, although it has been suggested to be a good method and has been previously applied for the *Arabidopsis*–*M. javanica* interaction system (Cabrera et al. 2015; Diaz-Manzano et al. 2018). It has been recently reported that positions of NFSs caused by *Heterodera avenae* relative to xylem vessels differ between susceptible and resistant wheat (Levin et al. 2021), implying that locations and architecture of NFSs might affect a functional efficiency facilitating nematode development and reproduction. In addition, different architectures among the three types of *M. graminicola*-caused NFSs might lead to a different parasitism ability of *M. graminicola* on rice. Noteworthy, the type I of *M. graminicola*-caused NFSs on rice that shows a similar size for its lateral and longitudinal areas presents a relatively larger surface than both types II and III, which could facilitate material and signal exchange between GCs and their neighboring cells (Bartlem et al. 2014; Kyndt et al. 2016; Xu et al. 2021). This might explain why type I is the dominant architectural type of NFSs in rice galls caused by *M. graminicola* and type II at later parasitic J2 stage transforms into type I at J3 stage. Developmental differences among type III and the types II and I were not assessed due to the extremely limited number of type III at each developmental stage.

Nevertheless, a description of the expansion progression of NFSs can help us to decipher their complex developmental mechanism. Here, the differences in lateral and longitudinal areas as well as volume of *M. graminicola*-caused NFSs on rice among the developmental stages analyzed were characterized for the first time. We observed that the lateral and longitudinal expansion of NFSs caused by *M. graminicola* stopped at later parasitic J2 stage and at J4 stage, respectively, indicating the expansion of *M. graminicola*-caused NFSs arrests at J4 stage. Polarity of GCs is also evidenced by the differential deposition of cell wall thickening seen by variance in wall labyrinths development (Berg et al. 2008). Offler et al. (2003) suggested that particular extracellular signals might be the cause of the polarized deposition during cell wall thickening (Offler et al. 2003). Remarkably, *M. graminicola*-caused NFSs on rice at both the non-parasitic J3 and J4 stages still undergo polarized expansion and an increase of GC cytoplasm density. The J3 and J4 stages of *M. graminicola* do not have a stylet. The stylet of the J2 sticks to the cuticle during molting, and the J3 and J4 stay enclosed in the lost cuticles. Thus, during this short molting period effector secretions as well as feeding do not occur during J3 and J4 stages. However, other effector molecules could be secreted by the nematode amphids and hypodermis (Eves-van den Akker et al. 2014; Rehman et al. 2016; Zhao et al. 2019). In addition, it cannot be ruled out that the polarized expansion of *M. graminicola*-caused NFSs on rice during J3 and J4 stages might also be orchestrated by extracellular signals derived from their neighboring cells besides *M. graminicola* themselves. Cell wall-modifying proteins secreted by sedentary parasitic nematodes and those from host plants recruited by plant parasitic nematodes are reported to function in cell wall modifications of NFSs (Goellner et al. 2001; Hewezi et al. 2008; Lichocka and Golinowski 2008; Ji et al. 2013; Wieczorek et al. 2014). It is reasonable to postulate that the polarized expansion progression of *M. graminicola*-caused NFSs on rice is accompanied by polarized depositions of proteins leading to cell wall modifications in GCs.

It is widely believed that karyokinesis without cytokinesis greatly contributes to GC formation and development, and plant genes involved in this process have been reported (de Almeida-Engler et al. 2012; Vieira et al. 2013, 2014; Cabral et al. 2020). Recognition of the developmental progression of GCs is addressed here and will contribute to the understanding on how feeding sites further develop. We observed an accumulation of nuclei around *M. graminicola* heads at early parasitic J2 stage and neighboring cells around their heads apparently undergo more intense division. A similar observation has been made in both *M. incognita*- and *M. javanica*-caused GCs on *Impatiens balsamina* and the authors believed that this facilitated GC expansion (Jones and Payne 1978). Changes in GC cytoplasm density have been previously investigated (Berg et al. 2008), providing valuable information to elucidate GC developmental progression. But analyses of cytoplasm densities of NFSs covering all their developmental stages as done here give a more complete and comprehensive view of their development. Aiming to perform a deeper evaluation of the parasitic J2 stage, as a key initial stage of GC formation, it was artificially classified into four substages according to the difference in GC cytoplasm density and morphological features of nematodes. Noteworthy, an extensive investigation of difference in cytoplasm density of GCs at early parasitic J2 stage was conducted by artificially dividing this stage into three substages, the early, middle and later of early parasitic J2 stage. Strikingly, it was found that cytoplasmic density of GCs significantly increased at the middle of early parasitic J2 stage, then followed by a distinct decrease later in the early parasitic J2 stage. A similar result has been reported in *M. incognita*-caused GCs on *Arabidopsis*, where a dense cytoplasm surrounding the young parasitic J2 was characterized during the initial NFS activation (Vieira et al. 2014). A distinct decrease of cytoplasm density of *M. graminicola*-caused GCs on rice later in the early parasitic J2 stage is most likely due to the formation of large central vacuoles in the GCs at this stage, because large central vacuoles are always observed in young GCs, and these large vacuoles are fragmented into smaller ones in mature GCs (Jones and Dropkin 1975; Hammes et al. 2005; Sobczak and Golinowski 2009; Escobar et al. 2015). Increased density of cytoplasm resulted in stronger green fluorescence in GCs, and we showed here that cytoplasm density in *M. graminicola*-caused GCs on rice increases after the midpoint of early parasitic J2 stage showing highest fluorescence during adult female stage. Escobar et al. (2015) suggested that GCs with high density of cytoplasm is a marked feature of mature GCs. The increased cytoplasm density of GCs might be implicated in the overall increase in metabolic activity required for nematode development ensuring sufficient nutrient supply (de Almeida-Engler and Gheysen 2013; Bartlem et al. 2014; de Almeida-Engler et al. 2015).

Overall, our data suggest the formation of three types of NFSs by *M. graminicola* on rice and polarized expansion until the J4 stage. In addition, NFS expansion does not synchronize with the maturation of NFSs with the expansion being completed before full NFS maturation.

### *Author contribution statement*

All the authors contributed to the study conception and design. Material preparation, data collection and analysis were performed by YN, LX, GW, JA-E, GG, DP, XX, and WH. The first draft of the manuscript was written by GW, JA-E, and GG, and all the authors commented on previous versions of the manuscript. All the authors read and approved the final manuscript.

## Supplementary Information

Below is the link to the electronic supplementary material.

Supplementary file1 (DOCX 15 KB)

**Fig. S1** Correlation between the largest expanded areas of *M. graminicola*-caused NFSs on rice and their volumes at different stages. Regression equations of the largest expanded areas of *M. graminicola*-caused NFSs on rice and their volumes at later parasitic J2 (p-J2+) (**a**), J3 (**b**), J4 (**c**) and adult female (adult, **d**) stages were established. Intercept mathematics was set to 0.0 for all the regression equations. Both the regression equations (*y*) and correlation indexes (*R*^2^) were shown at the upper left of the graphs. (TIF 6723 KB)

**Fig. S2** Serial sections of *M. graminicola*-caused GCs at the early of early parasitic J2 stage. Serial sections of *M. graminicola*-caused GCs at the early of early parasitic J2 stage on rice were taken from an entire gall by using confocal microscopy. The developmental stage of the GCs was identified according to the morphology of the feeding nematode associated. Voxel depth = 2.79 μm, and bar = 50.00 μm. (TIF 7738 KB)

**Fig. S3** Serial sections of *M. graminicola*-caused GCs at the middle of early parasitic J2 stage. Serial sections of *M. graminicola*-caused GCs at the middle of early parasitic J2 stage on rice were taken from an entire gall by using confocal microscopy. The developmental stage of the GCs was identified according to the morphology of the feeding nematode associated. Voxel depth = 1.40 μm, and bar = 50 μm. (TIF 2714 KB)

**Fig. S4** Serial sections of *M. graminicola*-caused GCs at the later of early parasitic J2 stage. Serial sections of a *M. graminicola*-caused GCs at later of early parasitic J2 stage on rice were taken from an entire gall using confocal microscopy. The developmental stage of the GCs was identified according to the morphology of the feeding nematode associated. Voxel depth = 1.44 μm, and bar = 50 μm (TIF 8751 KB)

**Fig. S5** Comparison of the volume between *M. graminicola*-caused GCs with high and low GC cytoplasm density at early parasitic J2 stage. Volumes of *M. graminicola*-caused NFSs with high and low GC cytoplasm density were compared at early parasitic J2 stage. Values are means ± SE. ***Indicate significant differences (Student’s *t* test) at *P* < 0.001 (TIF 1535 KB)

**Fig. S6 **Serial sections of *M. graminicola*-caused GCs at later parasitic J2 stage. Serial sections of *M. graminicola*-caused GCs at later parasitic J2 stage on rice were taken from an entire gall by using confocal microscopy. The developmental stage of the GCs was identified according to the morphology of the feeding nematode associated. Voxel depth = 1.85 μm, and bar = 50 μm (TIF 4066 KB)

**Fig. S7** Serial sections of *M. graminicola*-caused GCs at J3 stage. Serial sections of *M. graminicola*-caused GCs at J3 stage on rice were taken from an entire gall using confocal microscopy. The developmental stage of the GCs was identified according to the morphology of the feeding nematode associated. Voxel depth = 1.50 μm, and bar = 50 μm (TIF 14308 KB)

**Fig. S8** Serial sections of *M. graminicola*-caused GCs at J4 stage. Serial sections of *M. graminicola*-caused GCs at J4 stage on rice were taken from an entire gall by using confocal microscopy. The developmental stage of the GCs was identified according to the morphology of the feeding nematode associated. Voxel depth = 2.21 μm, and bar = 50 μm (TIF 12452 KB)

**Fig. S9** Serial sections of *M. graminicola*-caused GCs at adult female stage. Serial sections of *M. graminicola*-caused GCs at adult female stage on rice were taken from an entire gall by using confocal microscopy. The developmental stage of the GCs was identified according to the morphology of the feeding nematode associated. Voxel depth = 1.91 μm, and bar = 50 μm (TIF 10072 KB)

**Video S1 **Three-dimensional structure of a type I *M. graminicola*-caused NFS on rice. Three-dimensional reconstruction of the type I of a *M. graminicola*-caused NFS at adult female stage on rice by using 3D View of the software Fiji. The complete serial sections were taken from an entire gall by confocal microscopy. Voxel depth = 1.96 μm, and bar = 50 μm. (AVI 138248 KB)

**Video S2 **Three-dimensional structure of a type II *M. graminicola*-caused NFS on rice. Three-dimensional reconstruction of the type II of a *M. graminicola*-caused NFS at adult female stage on rice by using 3D View of the software Fiji. The complete serial sections were taken from an entire gall by confocal microscopy. Voxel depth = 2.15 μm, and bar = 50 μm (AVI 138248 KB)

**Video S3** Three-dimensional structure of a type III *M. graminicola*-caused NFS on rice. Three-dimensional reconstruction of the type III of a *M. graminicola*-caused NFS at J3 stage on rice by using 3D View of the software Fiji. The complete serial sections were taken from an entire gall by confocal microscopy. Voxel depth = 1.67 μm, and bar = 50 μm (AVI 138248 KB)

## Data Availability

The datasets generated during and/or analyzed during the current study are available from the corresponding author on reasonable request.

## Abbreviations

GC: Giant cell
NFS: Nematode-feeding site
