# Supplementary material for: Morphological characterization reveals new insights into giant cell development of Meloidogyne graminicola on rice
Source: Planta. 2022 Feb 19;255(3):70. doi: 10.1007/s00425-022-03852-z (PMC8858295; doi:10.1007/s00425-022-03852-z)
Supplement: Supplementary file 1 — Supplementary file1 (DOCX 15 KB) [file 425_2022_3852_MOESM1_ESM.docx]

Table S1. Numbers of *M. graminicola*-caused NFSs on rice analyzed at each developmental stage

| Developmental stages | Numbers of *M. graminicola*-caused NFSs on rice assayed | | |
| --- | --- | --- | --- |
|  | Lateral area | Longitudinal area | Volume |
| Early parasitic J2 (p-J2) | / | / | 35 |
| Later parasitic J2 (p-J2+) | 22 | 22 | 22 |
| J3 | 32 | 32 | 32 |
| J4 | 27 | 27 | 27 |
| Adult female | 51 | 51 | 51 |

“/”, indicates the number of *M. graminicola*-caused NFSs on rice analyzed is zero.
