# Supplementary figures and images for: Morphological characterization reveals new insights into giant cell development of Meloidogyne graminicola on rice
Source: Planta. 2022 Feb 19;255(3):70. doi: 10.1007/s00425-022-03852-z (PMC8858295; doi:10.1007/s00425-022-03852-z)

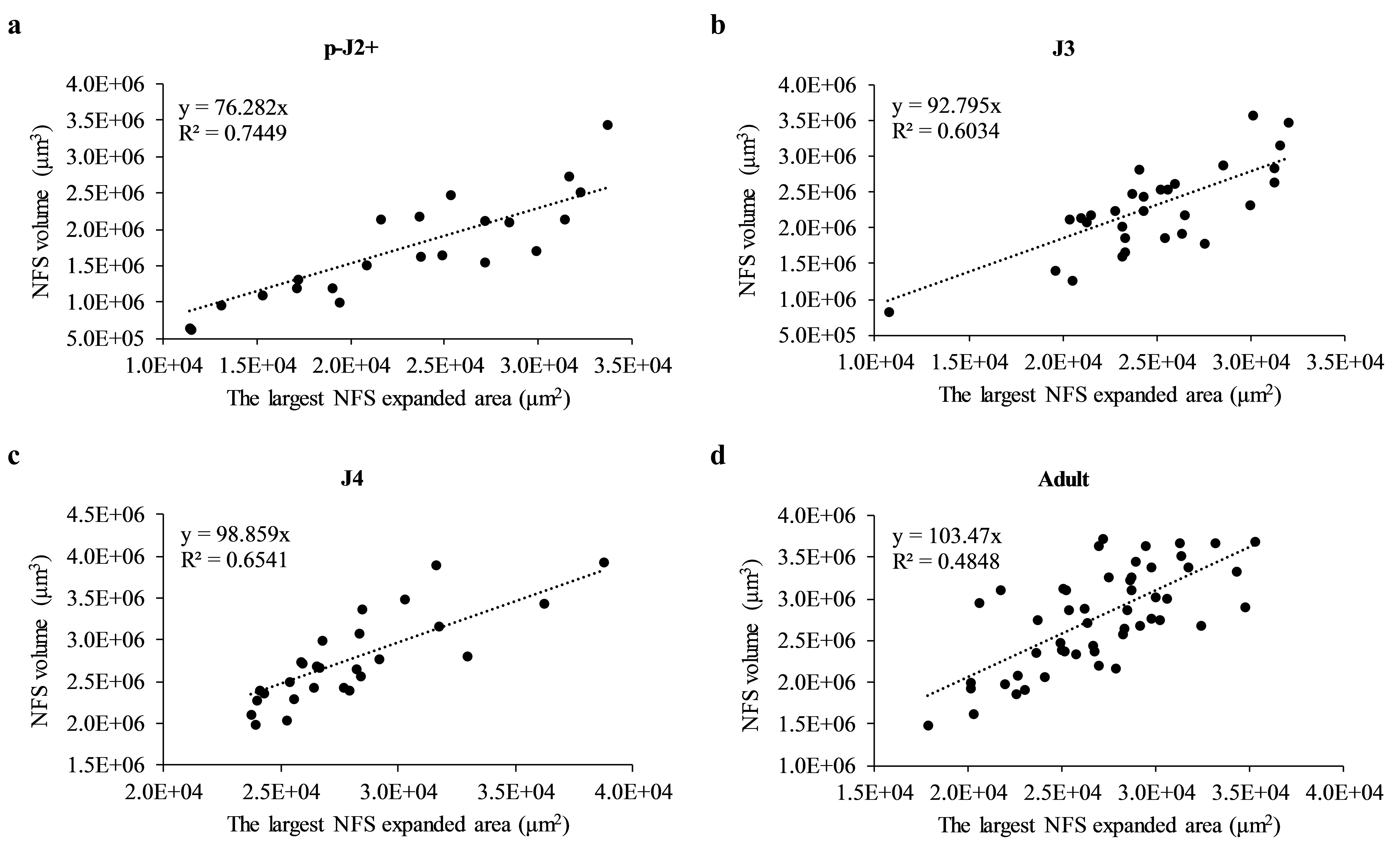

Supplement: Supplementary file 2 — Fig. S1 Correlation between the largest expanded areas of M. graminicola-caused NFSs on rice and their volumes at different stages. Regression equations of the largest expanded areas of M. graminicola-caused NFSs on rice and their volumes at later parasitic J2 (p-J2+) (a), J3 (b), J4 (c) and adult female (adult, d) stages were established. Intercept mathematics was set to 0.0 for all the regression equations. Both the regression equations (y) and correlation indexes (R2) were shown at the upper left of the graphs. (TIF 6723 KB) [file 425_2022_3852_MOESM2_ESM.tif]

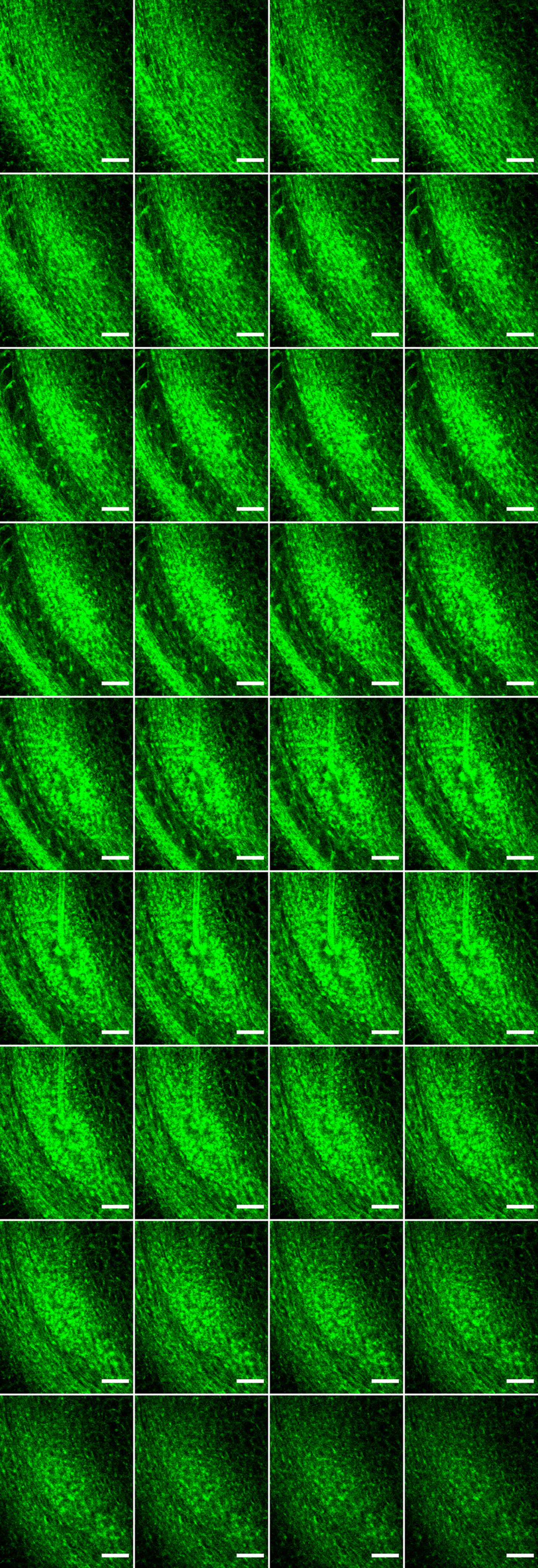

Supplement: Supplementary file 3 — Fig. S2 Serial sections of M. graminicola-caused GCs at the early of early parasitic J2 stage. Serial sections of M. graminicola-caused GCs at the early of early parasitic J2 stage on rice were taken from an entire gall by using confocal microscopy. The developmental stage of the GCs was identified according to the morphology of the feeding nematode associated. Voxel depth = 2.79 μm, and bar = 50.00 μm. (TIF 7738 KB) [file 425_2022_3852_MOESM3_ESM.tif]

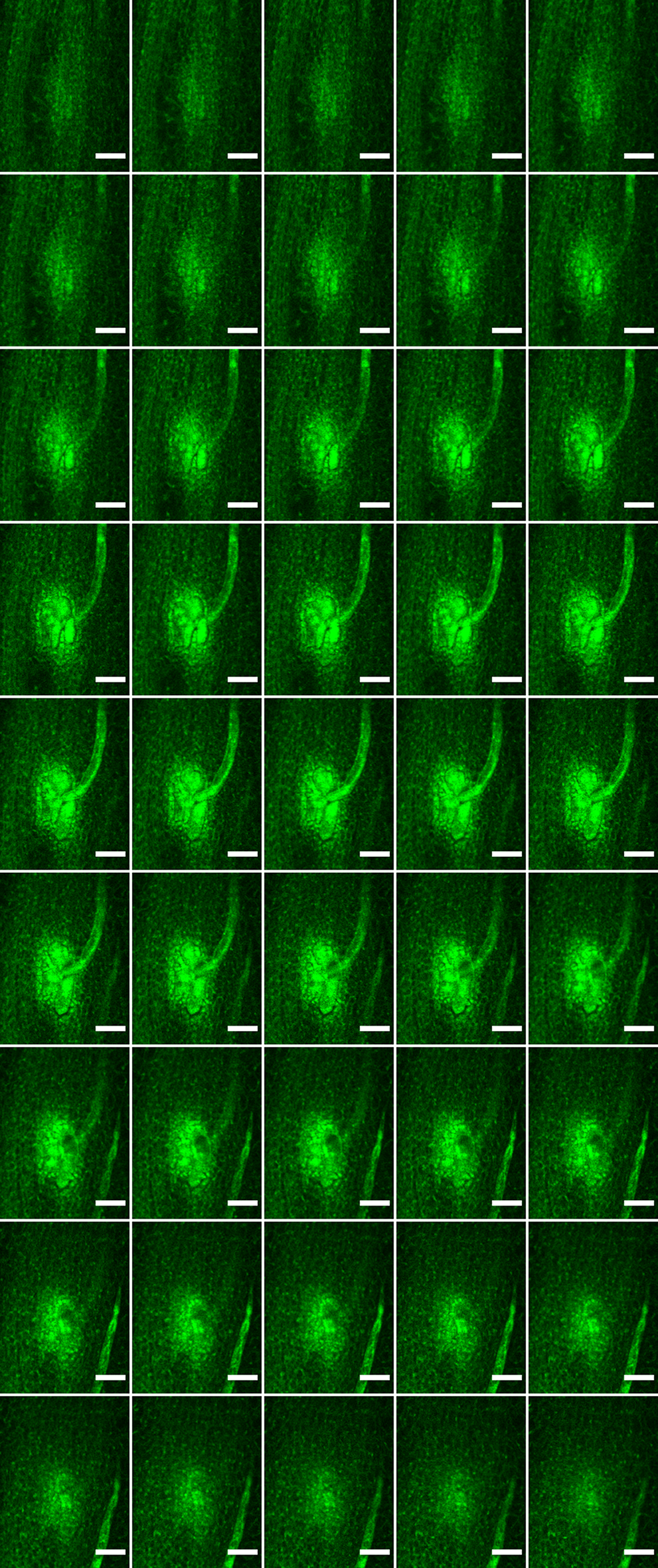

Supplement: Supplementary file 4 — Fig. S3 Serial sections of M. graminicola-caused GCs at the middle of early parasitic J2 stage. Serial sections of M. graminicola-caused GCs at the middle of early parasitic J2 stage on rice were taken from an entire gall by using confocal microscopy. The developmental stage of the GCs was identified according to the morphology of the feeding nematode associated. Voxel depth = 1.40 μm, and bar = 50 μm. (TIF 2714 KB) [file 425_2022_3852_MOESM4_ESM.tif]

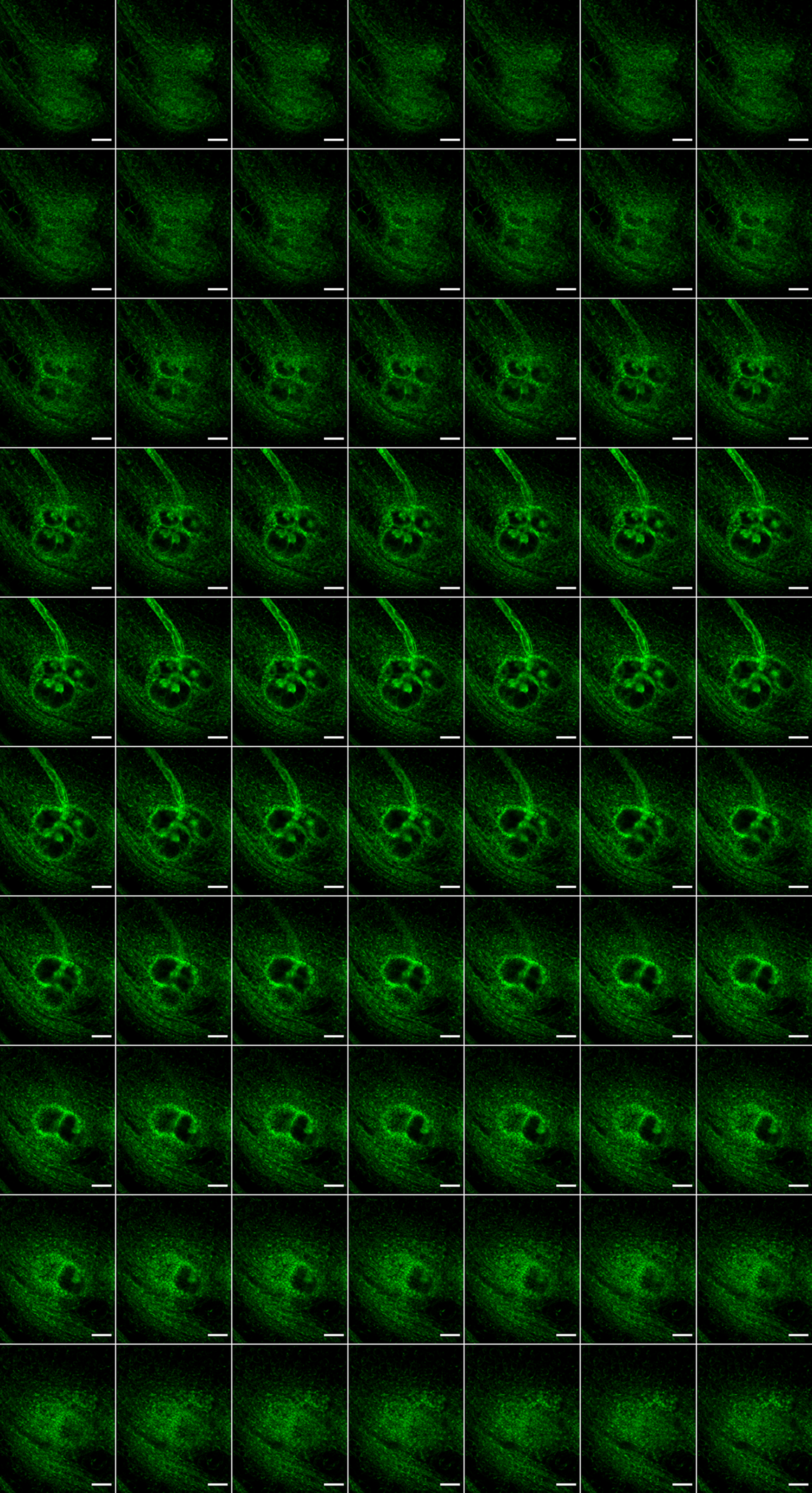

Supplement: Supplementary file 5 — Fig. S4 Serial sections of M. graminicola-caused GCs at the later of early parasitic J2 stage. Serial sections of a M. graminicola-caused GCs at later of early parasitic J2 stage on rice were taken from an entire gall using confocal microscopy. The developmental stage of the GCs was identified according to the morphology of the feeding nematode associated. Voxel depth = 1.44 μm, and bar = 50 μm (TIF 8751 KB) [file 425_2022_3852_MOESM5_ESM.tif]

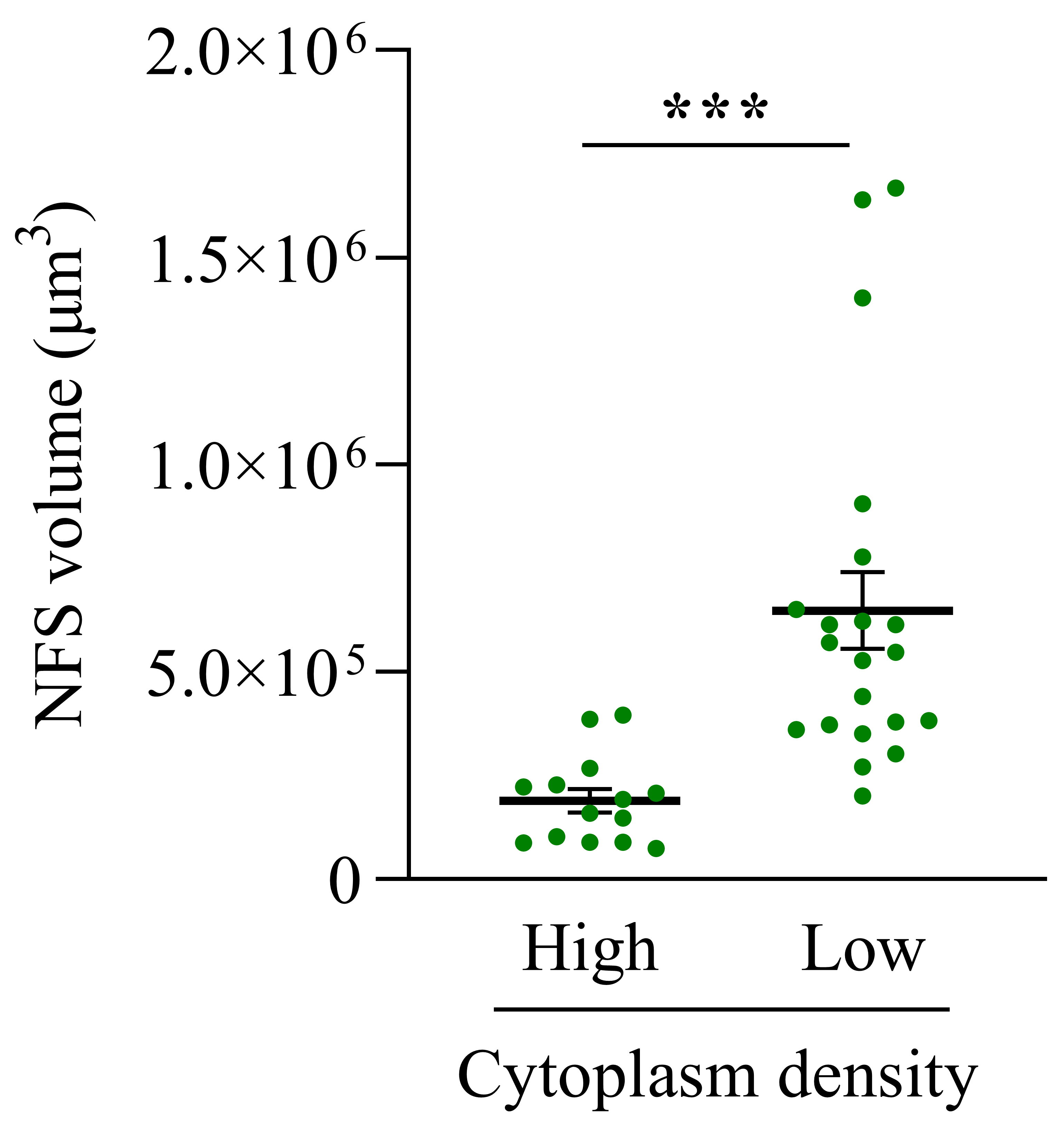

Supplement: Supplementary file 6 — Fig. S5 Comparison of the volume between M. graminicola-caused GCs with high and low GC cytoplasm density at early parasitic J2 stage. Volumes of M. graminicola-caused NFSs with high and low GC cytoplasm density were compared at early parasitic J2 stage. Values are means ± SE. ***Indicate significant differences (Student’s t test) at P < 0.001 (TIF 1535 KB) [file 425_2022_3852_MOESM6_ESM.tif]

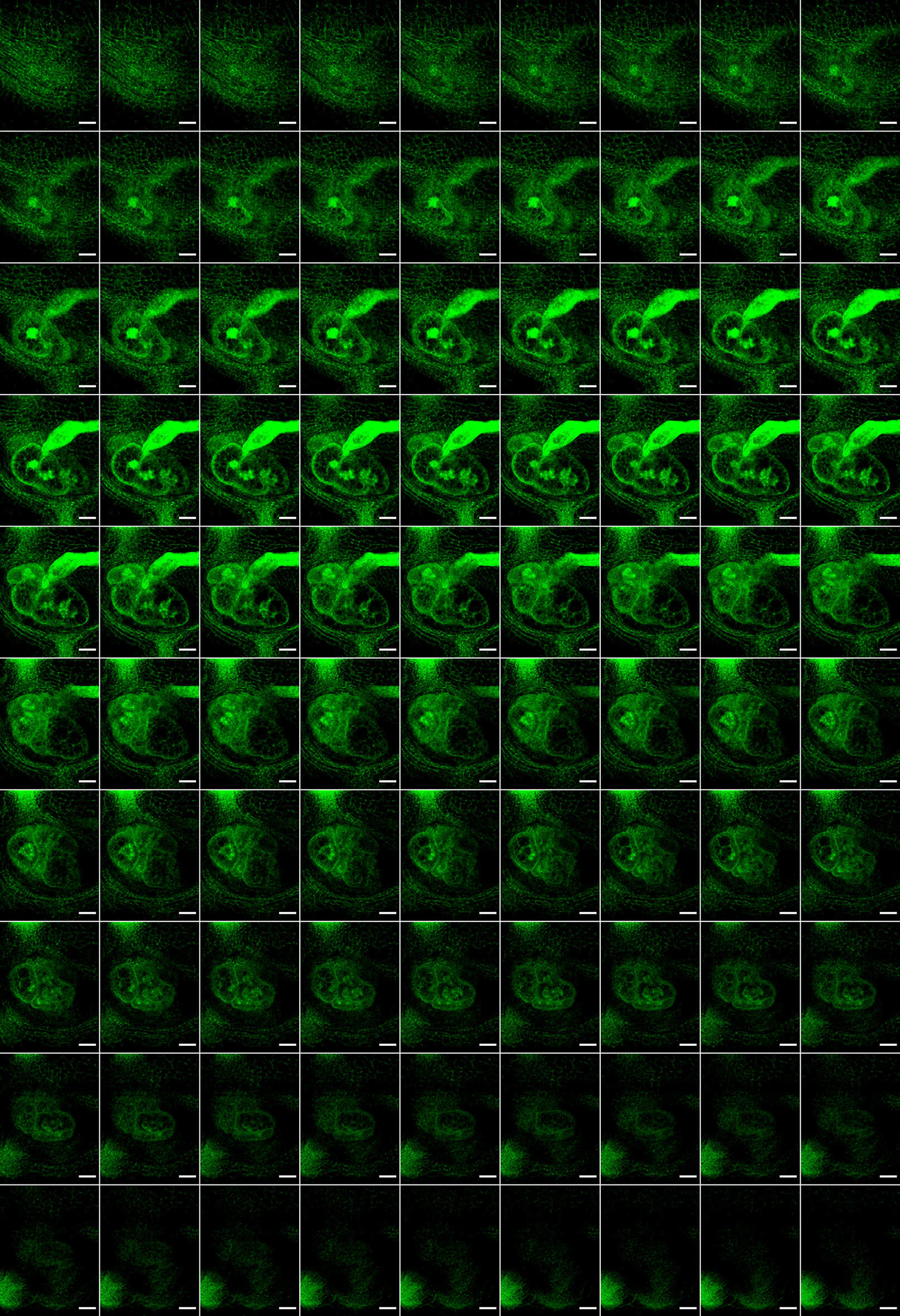

Supplement: Supplementary file 7 — Fig. S6 Serial sections of M. graminicola-caused GCs at later parasitic J2 stage. Serial sections of M. graminicola-caused GCs at later parasitic J2 stage on rice were taken from an entire gall by using confocal microscopy. The developmental stage of the GCs was identified according to the morphology of the feeding nematode associated. Voxel depth = 1.85 μm, and bar = 50 μm (TIF 4066 KB) [file 425_2022_3852_MOESM7_ESM.tif]

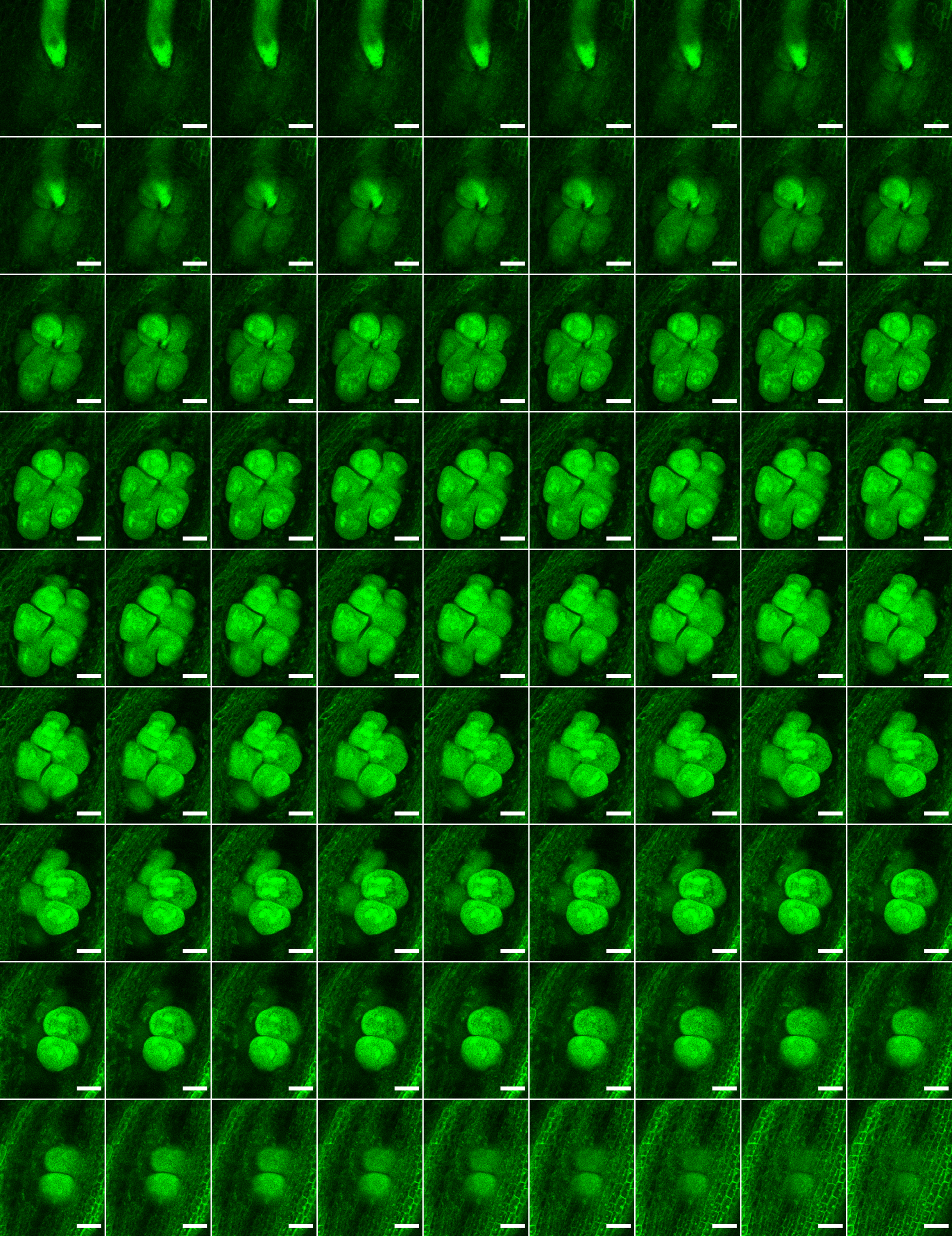

Supplement: Supplementary file 8 — Fig. S7 Serial sections of M. graminicola-caused GCs at J3 stage. Serial sections of M. graminicola-caused GCs at J3 stage on rice were taken from an entire gall using confocal microscopy. The developmental stage of the GCs was identified according to the morphology of the feeding nematode associated. Voxel depth = 1.50 μm, and bar = 50 μm (TIF 14308 KB) [file 425_2022_3852_MOESM8_ESM.tif]

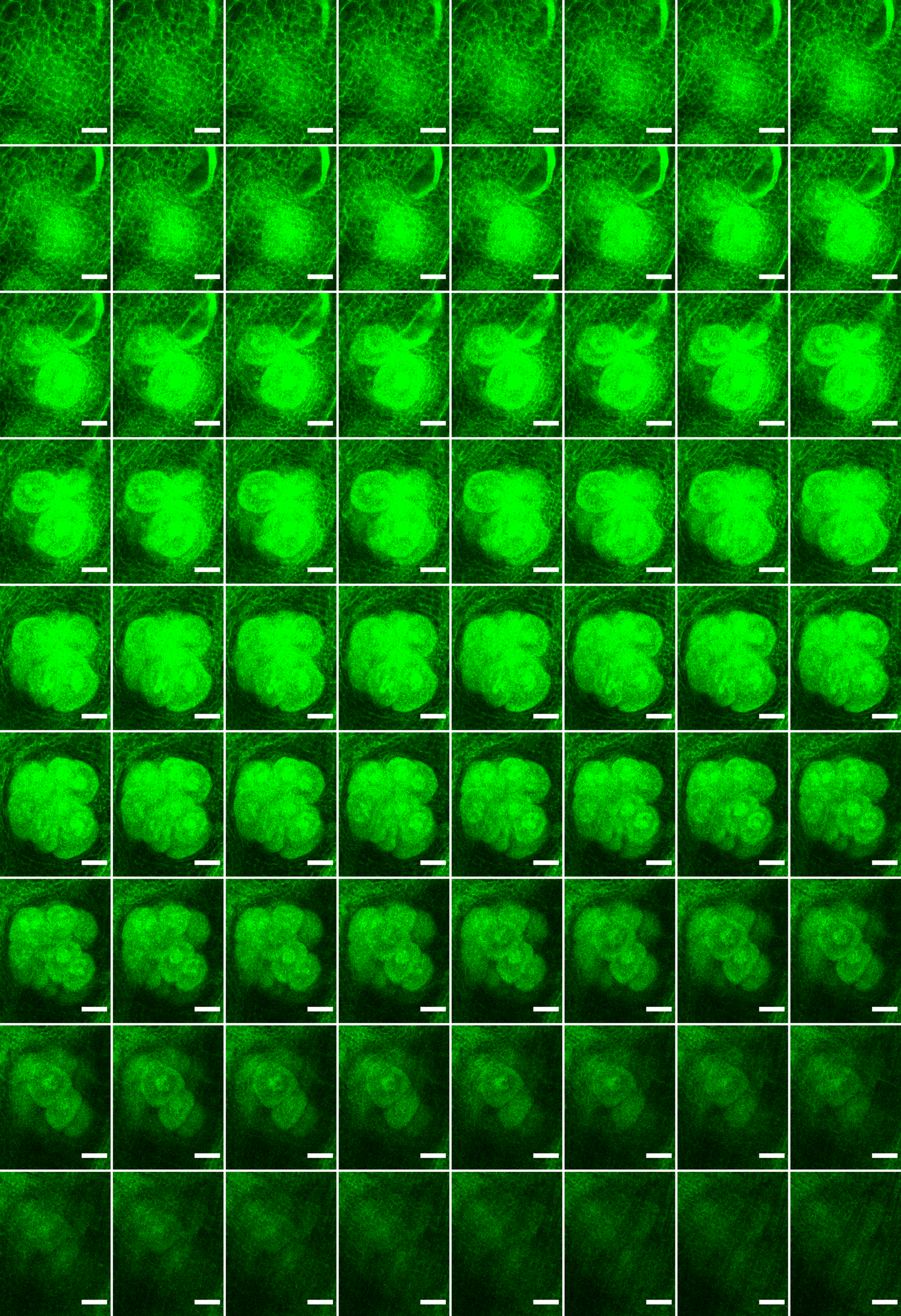

Supplement: Supplementary file 9 — Fig. S8 Serial sections of M. graminicola-caused GCs at J4 stage. Serial sections of M. graminicola-caused GCs at J4 stage on rice were taken from an entire gall by using confocal microscopy. The developmental stage of the GCs was identified according to the morphology of the feeding nematode associated. Voxel depth = 2.21 μm, and bar = 50 μm (TIF 12452 KB) [file 425_2022_3852_MOESM9_ESM.tif]

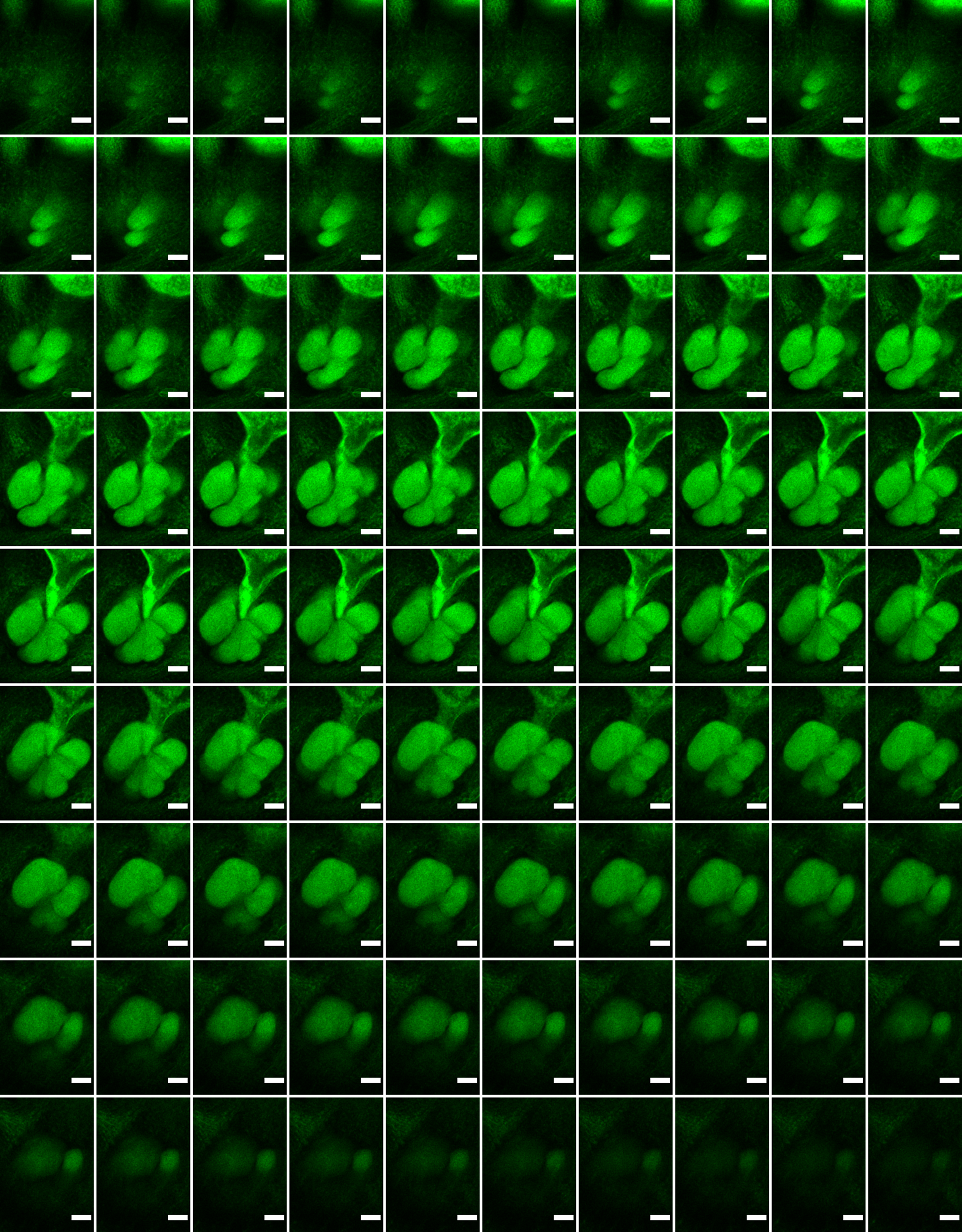

Supplement: Supplementary file 10 — Fig. S9 Serial sections of M. graminicola-caused GCs at adult female stage. Serial sections of M. graminicola-caused GCs at adult female stage on rice were taken from an entire gall by using confocal microscopy. The developmental stage of the GCs was identified according to the morphology of the feeding nematode associated. Voxel depth = 1.91 μm, and bar = 50 μm (TIF 10072 KB) [file 425_2022_3852_MOESM10_ESM.tif]
